# A Multi-Strategy Sequencing Workflow in Inherited Retinal Dystrophies: Routine Diagnosis, Addressing Unsolved Cases and Candidate Genes Identification

**DOI:** 10.3390/ijms21249355

**Published:** 2020-12-08

**Authors:** Marta Martín-Sánchez, Nereida Bravo-Gil, María González-del Pozo, Cristina Méndez-Vidal, Elena Fernández-Suárez, Enrique Rodríguez-de la Rúa, Salud Borrego, Guillermo Antiñolo

**Affiliations:** 1 Department of Maternofetal Medicine, Genetics and Reproduction, Institute of Biomedicine of Seville, University Hospital Virgen del Rocío/CSIC/University of Seville, 41013 Seville, Spain; marta.martin.sanchez@juntadeandalucia.es (M.M.-S.); nereida.bravo@juntadeandalucia.es (N.B.-G.); maria.gonzalez@ciberer.es (M.G.-d.P.); cristina.mendez@juntadeandalucia.es (C.M.-V.); mariae.fernandez.suarez@juntadeandalucia.es (E.F.-S.); salud.borrego.sspa@juntadeandalucia.es (S.B.); 2 Centro de Investigación Biomédica en Red de Enfermedades Raras (CIBERER), 41013 Seville, Spain; 3 Department of Ophthalmology, University Hospital Virgen Macarena, 41013 Seville, Spain; enrique.rodriguezrua.sspa@juntadeandalucia.es; 4 Retics Patologia Ocular, OFTARED, Instituto de Salud Carlos III, 28029 Madrid, Spain

**Keywords:** next generation sequencing, inherited retinal dystrophies, genetic diagnosis, *WDFY3*, *CITED1*

## Abstract

The management of unsolved inherited retinal dystrophies (IRD) cases is challenging since no standard pipelines have been established. This study aimed to define a diagnostic algorithm useful for the diagnostic routine and to address unsolved cases. Here, we applied a Next-Generation Sequencing-based workflow, including a first step of panel sequencing (PS) followed by clinical-exome sequencing (CES) and whole-exome sequencing (WES), in 46 IRD patients belonging to 42 families. Twenty-six likely causal variants in retinal genes were found by PS and CES. CES and WES allowed proposing two novel candidate *loci* (*WDFY3* and a X-linked region including *CITED1*), both abundantly expressed in human retina according to RT-PCR and immunohistochemistry. After comparison studies, PS showed the best quality and cost values, CES and WES involved similar analytical efforts and WES presented the highest diagnostic yield. These results reinforce the relevance of panels as a first step in the diagnostic routine and suggest WES as the next strategy for unsolved cases, reserving CES for the simultaneous study of multiple conditions. Standardizing this algorithm would enhance the efficiency and equity of clinical genetics practice. Furthermore, the identified candidate genes could contribute to increase the diagnostic yield and expand the mutational spectrum in these disorders.

## 1. Introduction

Inherited Retinal Dystrophies (IRD) are a group of degenerative diseases characterized by loss of photoreceptors, resulting in visual impairment or blindness. These disorders have a high clinical and genetic heterogeneity, with different patterns of inheritance and more than 250 associated-genes so far [1] (RetNet, https://sph.uth.edu/retnet, last accessed 5 May 2020). For these reasons, making an accurate diagnosis of IRD has traditionally been a difficult and expensive task. However, the use of next generation sequencing (NGS) techniques has revolutionized the field of genetics and genomics and has been a great step forward in the molecular diagnosis of families with these mendelian diseases, which has resulted in a more precise clinical diagnosis and a better clinical management of patients [2,3,4].

Currently, there is a wide range of NGS-applications and the choice of a particular approach depends mainly on the cost-benefit balance and the scope. In the clinical field, gene-panel sequencing (PS) is usually the first step for the detection of disease-causative genetic variants [5,6], however around 40% of cases remain unsolved [7,8]. The inclusion criteria of genes for the design of these panels are also of variable character, since, while some studies conceive panels with all known disease-associated genes [5], personalized strategies including only those *loci* affecting a particular population (population-specific panels) seem to be the most cost-efficient option [2] as the prevalence of causative genes and mutations have a strong population factor [9]. Nevertheless, the workflow for cases that remain unsolved after the application of this first strategy has not been fully defined either. Although whole genome sequencing (WGS) is the most complete NGS approach, its use in the clinical setting is very limited due to its high cost and the difficulty of handling large amounts of data [10]. Thus, other methods in which sequencing is restricted to a specific fraction of the genome, such as whole exome sequencing (WES) and clinical exome sequencing (CES), are commonly used to address the study of genetically undiagnosed cases.

Targeted sequencing of a large number of genes not limited to a specific condition allows carrying out a hypothesis-free approach with high capacity to establish new genotype–phenotype correlations. In this context, WES is a widely used high-performance strategy because most pathogenic mutations are located in exonic regions [11,12,13] and provides additional advantages for discovery purposes. Moreover, although several deep-intronic mutations have been reported in IRD cases [14,15], their contribution to the etiopathogenesis of the IRD in our cohort is unclear [16]. Therefore, sequencing all exons of the genome could be an efficient second step in the genetic diagnosis of IRD [17]. However, its translation to the healthcare setting is not always feasible due to its still high cost, data management challenges and the lack of all-in-one open access commercial bioinformatics solutions [18], reducing its use to high-resource centers and deep experienced departments. In this context, the need to look for alternative tools with greater accessibility and equity feasible to be transferred to the clinical practice led to the emergence of CES.

CES is defined as a reduced version of WES containing only disease-associated genes and can be performed using both commercial and customized methods, allowing a high flexibility. The efficiency of this approach compared to other NGS-strategies, as well as its role in a NGS-based workflow for genetic diagnosis, has not been well described yet. In some cases, CES has been used as the first diagnostic strategy instead of disease-specific gene panels [19] in order to avoid arduous designs and overcome the blurred phenotype limits among different conditions, while in others it has been used as a substitute for WES as a limited discovery tool [20]. Additionally, CES could also be applied as an intermediate tool between small panels and WES or WGS. 

In this study, 46 patients with IRD, belonging to 42 unrelated families, underwent a NGS-based workflow consisting of a first step of disease-specific PS, followed by CES and subsequent WES in the respective unsolved cases. This approach allowed us to evaluate the corresponding diagnostic yields of these techniques and their ability to expand the disease spectrum, as well as elucidating the relevance of each of them in the routine of both medical practice and clinical research. 

## 2. Results

### 2.1. Genetic Diagnosis and Screening Unsolved Cases by Panel Sequencing

Samples analyzed by PS showed a mean coverage of 654.8×, with 99.6% of covered bases and 96.6% of reads mapped on target. The genetic analysis led to the identification of 33 candidate mutations, of which seven were novel, in 22 families (Table 1). However, a full diagnosis was only achieved in 20 of these families, since potential splicing variants in *WHRN* (c.1417-8G>A) and *PDE6B* (c.1593A>T) were found to segregate in Families 14 and 35, respectively, but functional studies could not be conducted and would be needed to assess their pathogenicity. Remarkably, the intrafamilial variability observed in Family 35 could not be explained based solely on the *PDE6B* variants and, therefore, additional genetic causes and/or phenotypic modifiers should be considered. Interestingly, in seven cases initially classified as RP, genetic results allowed us to ascertain an accurate clinical diagnosis (Families 2, 3, 4, 10, 24, 25 and 26) (Table S1). Altogether, this approach allowed us to fully reveal the underlying genetic defect in 20 cases and select 22 families without an accurate genetic diagnosis for further studies.

### 2.2. Detection of Pathogenic Mutations in Retinal Genes by Clinical Exome Sequencing

**Figure 1:**
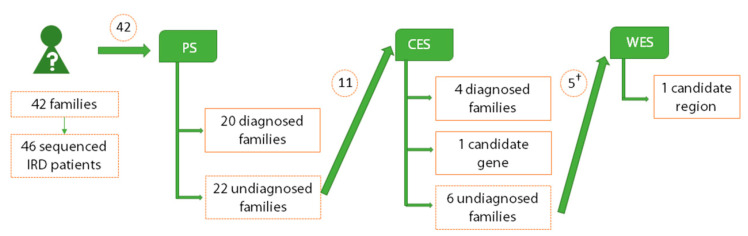
Summary of the applied pipeline and global diagnostics results. The application of panel sequencing (PS) allowed the genetic diagnosis of 20 of the 42 families included in the study. Afterwards, 11 of the 22 undiagnosed families were analyzed using a clinical exome sequencing (CES) approach, which allowed the detection of the genetic cause in four additional families and the identification of a potential candidate gene in another family. Finally, whole-exome sequencing (WES) of five cases without a genetic diagnosis after CES led to the detection of a region potentially including a novel IRD-associated gene. † In four out of five families that underwent WES, an alternative affected individual was sequenced instead of the corresponding index patients, making a total of 46 sequenced IRD patients.

DNA samples from 11 of the 22 unsolved cases met the quality and pedigree established criteria and were subjected to CES (Figure 1), resulting in a mean coverage of 591.5×, with 99.9% of covered bases and 75.3% of reads mapped on target. Applying this method, a full diagnosis was achieved in four out of 11 individuals (Table 2). In three of the cases (Families 17, 37 and 40), causative mutations could not be previously identified since the mutated genes were not included in the custom panel (*MFRP*, *FAM161A* and *RP1L1*, respectively). The fourth solved case (Family 36) harbored the hemizygous mutation c.2655_2656del; p.(Glu886Glyfs*192) in the ORF15 of *RPGR*, a region included in the design of the previous panel but not covered due to its repetitive nature (Figure S1), which made it difficult to be captured and sequenced. Thus, in this strategy a depth of 8× was obtained for this position in the index patient, allowing variant calling. Sanger sequencing showed that both sisters of the index patient and his mother, all of them presumably asymptomatic, also harbored the mutation in heterozygous state, while his affected nephew bore the same hemizygous mutation. After clinical examination, it was revealed that all female carriers showed signs of mild/moderate retinal impairment, while the nephew presented a severe form of RP reminiscent of the phenotype observed in the index patient.

### 2.3. Identification of Novel Candidate Genes by Clinical Exome Sequencing

**Figure 2:**
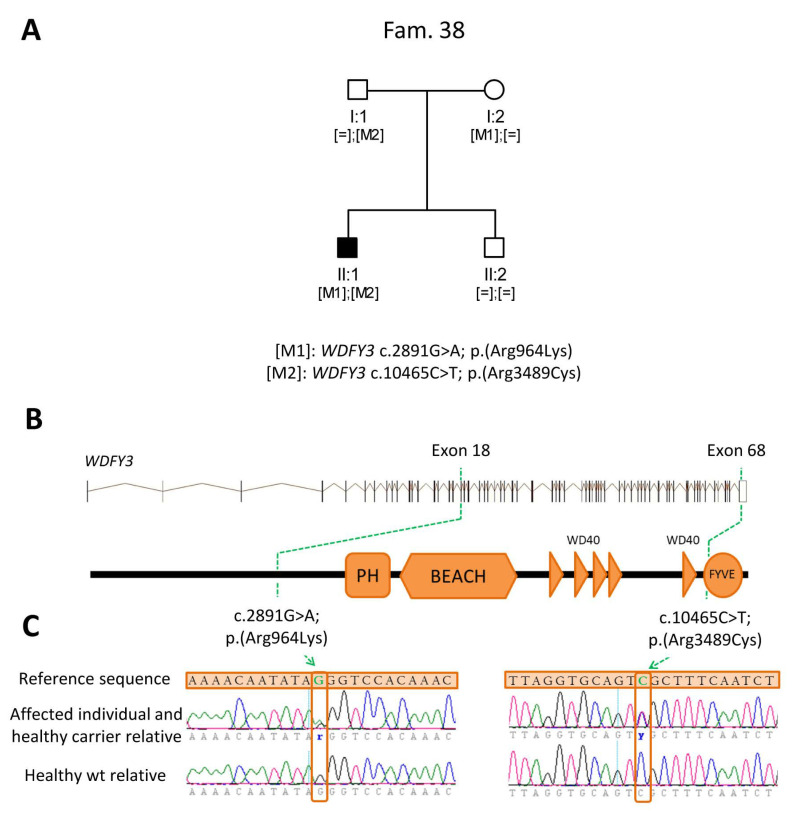
Pedigree of candidate variants detected in Family 38 and schematic representation of the identified *WDFY3* mutations. (**A**) Pedigree of Family 38 and segregation study of the detected variants in *WDFY3*. (**B**) Genomic and protein location of the segregating variants in *WDFY3* (NM_014991), which is composed of 68 exons (vertical lines). The protein depiction was performed according to Uniprot, showing five beta-transducin repeats (WD40) and three different domains: pleckstrin homology (PH), beige and Chediak–Higashi (BEACH) and Fab1, YOTB, Vac1, and EEA1 (FYVE) zinc finger. The location of the identified mutations is stated with a broken green line. (**C**) Electropherograms illustrating the detected *WDFY3* variants in heterozygous state and the wild type sequence.

In one of the families that underwent CES (Family 38), two candidate variants (Figure 2A) were identified in *WDFY3* (Figure 2B), a gene not associated with IRD so far. These variants (c.2891G>A, p.(Arg964Lys) and c.10465C>T, p.(Arg3489Cys)) could be acting in compound heterozygosity to generate the disease in a recessive manner (Figure 2C). In addition, the currently available information for this gene and the identified mutations (Table 3), led to consider *WDFY3* as candidate to be disease-causative and selected for further studies to elucidate their involvement in the development of IRD in this family.

### 2.4. Identification of Novel Candidate Regions by Whole Exome Sequencing

**Figure 3:**
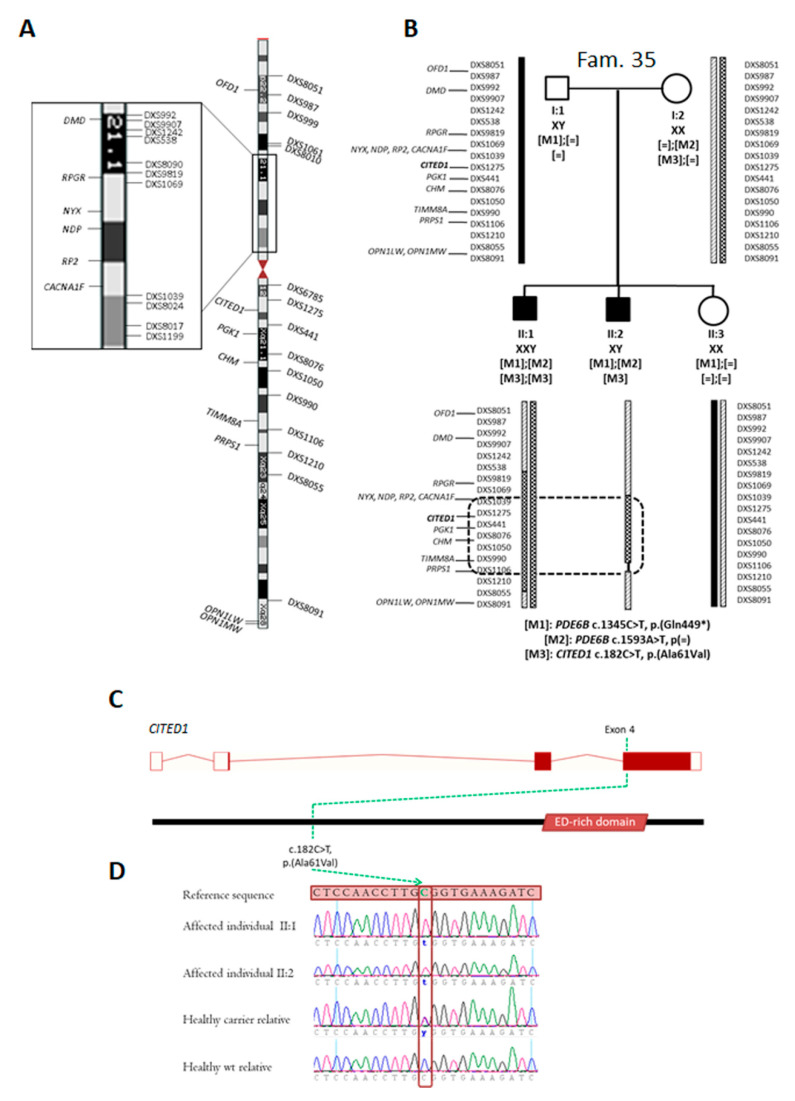
Segregation studies, linkage analyses and schematic representation of candidate variants detected in Family 35. (**A**) Diagram of chromosome X, showing the location of the used STR markers, X-linked IRD genes and the candidate gene CITED1. The region including RPGR is highlighted for clarity reasons. (**B**) Linkage analysis and segregation studies. Segregation analyses of PDE6B (M1 and M2) and CITED1 (M3) variants, detected by panel sequencing and whole exome sequencing, respectively, are shown. X-linked IRD associated genes and CITED1 (in bold letters) are indicated. Only those microsatellite markers (18 out of 26) adjacent to IRD genes or recombination points are displayed. Paternal X-chromosome is colored in black, while maternal X-chromosomes are marked by striped and checkered patterns, respectively. In individual II:2, linkage results did not allow determining whether the recombination between DXS990 and DXS1210 markers occurs upstream or downstream of the DXS1106 marker, which is shown with a thin black line. The black doted box contains the maximum common region between affected brothers, not shared by their healthy sister. (**C**) Genomic and protein location of the segregating variant in the CITED1 gene, which is composed of four exons (NM_001144885). Coding exons are shown as filled boxes while unfilled boxes reflect UTRs. The protein depiction was performed according to Uniprot, showing the glutamic acid/aspartic acid-rich domain (ED-rich domain) that characterizes the CITED family. The location of the identified CITED1 mutation is stated with a broken green line. (**D**) Electropherograms illustrating the detected CITED1 variant both in hemizygous/homozygous (affected individuals) and heterozygous (carrier female) state, as well as the wild type sequence.

Furthermore, according to quality assessment, five of the six cases remained unsolved after CES were selected for WES studies (Figure 1), resulting in a 74% of reads mapping on target, a 97.8% of covered bases and a mean coverage of 108.5× per sample. This strategy allowed us to initially propose a candidate region for one of the families. In individual II:2 of Family 35, no potential causal variants, beyond previously found *PDE6B* changes, or phenotype modifier alleles were detected following our prioritization criteria. To clarify the clinical variability between both brothers and, considering that one of them was affected with Klinefelter syndrome (47, XXY), a linkage analysis using STR markers along the entire X-chromosome was carried out (Figure 3A and Table S2). Linkage analysis led to the identification of a common region in both affected brothers (II:1 and II:2) in homozygous and hemizygous state, respectively (Figure 3B). This shared region included eight previously described IRD-associated genes (*NYX*, *NDP*, *RP2*, *CACNA1F*, *PGK1*, *CHM*, *TIMM8A* and *PRPS1*), but no coding variants were detected in either of those genes. A comprehensive study of the mutations in this X-linked common region using lax filters (not using control genotype filter and ACMG criteria) allowed the detection of a novel hemizygous mutation in *CITED1* (c.182C>T, p.(Ala61Val)) (Figure 3C,D), discarded in the initial analysis due to the presence of two individuals with this hemizygous mutation in GnomAD (Table 3). 

### 2.5. Retinal Expression of Candidate Genes

**Figure 4:**
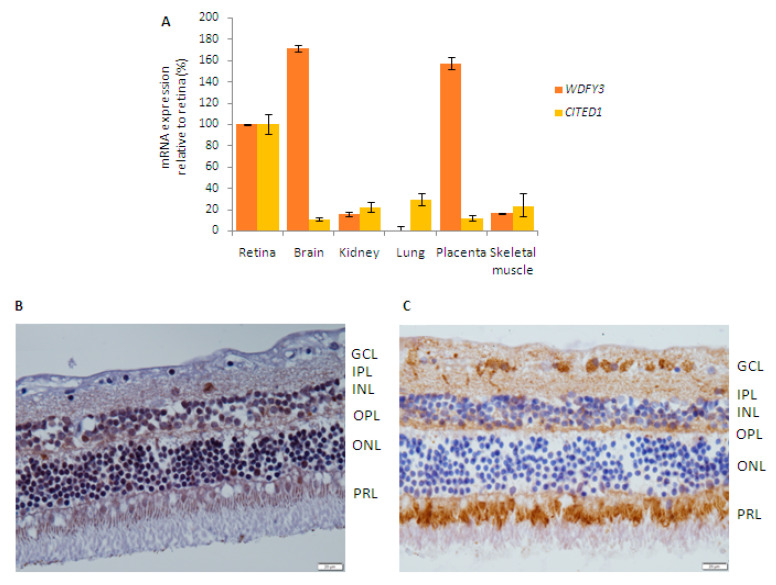
Expression analysis and localization of candidate genes. (**A**) Relative quantification of candidate genes *WDFY3* (orange) and *CITED1* (yellow) mRNA levels in different human tissues by real-time PCR. Data are represented as mean ± standard deviation. (**B**) Immunohistochemical staining of CITED1 in human retina sections (image captured at 60x magnification). Expression of CITED1 was observed in the photoreceptor layer (PRL), the outer nuclear layer (ONL) and the inner nuclear layer (INL). (**C**) Immunohistochemistry of WDFY3 in human retina sections, showing expression of WDFY3 in the PRL and ganglion cell layer (GCL). Results are shown at 60x magnification, with a 20 µm microscopic length as standard length. IPL, inner plexiform layer; OPL, outer plexiform layer. Both images present a different white balance, as micrographs were taken at different times.

To further study the involvement of candidate genes with segregating variants in the development of IRD, we first investigated their expression levels in cDNA from retina and other human tissues. The two genes analyzed (*WDFY3* and *CITED1*) were expressed in retinal tissue (Figure 4A). In the case of WDFY3 mRNA, the highest expression levels were found in the brain and placenta followed by retina. On the other hand, CITED1 mRNA levels were the highest in retina compared to the rest of analyzed tissues. 

The expression pattern of candidate genes was also investigated by immunohistochemical analyses using healthy human retina sections. On the one hand, specific immunolabeling with the CITED1 antibody was detected in rod and cone photoreceptors as well as in the inner and outer nuclear layers (Figure 4B). On the other hand, specific immunolabeling with the WDFY3 antibody was observed in rod and cone photoreceptors and in the ganglion cell layer (Figure 4C). CITED1 and WDFY3 immunoreactivity could not be assessed in the retinal pigment epithelium due to the heavily pigmented nature of these cells. 

### 2.6. Comparison among Applied Sequencing Methods

Sequencing of DNA samples with various approaches resulted in datasets whose main differences focused on data quality, number of variants, required time for tertiary analysis, diagnostic rate and costs (Table S3). Concerning quality, CES offered the highest percentage of covered bases, while PS surpassed it in mean coverage and reads mapped on target. Regarding the number of variants, as expected, this was greater as the target region increased. For this reason, less analytical efforts were necessary with panels whereas no notable differences were found between CES and WES. The comparison of the diagnostic yield showed that gene panels covered the causative genes in 87.5% of the total diagnosed cases and that, although both CES and WES could have detected all mutations in known IRD genes, only WES was able to identify all additional variants in new candidate genes. Additionally, panel sequencing had the lowest costs in sequencing a sample, with the same depth as other methods. Increasing the number of regions comprehended in each study also elevated the costs of each approach, which made WES the most expensive strategy. 

## 3. Discussion

In this study, we performed a targeted sequencing pipeline in order to define an efficient NGS-based workflow for the diagnosis of IRD cases. This strategy was applied to a total of 42 IRD families, which led to the identification of potential causative variants in known retinal genes and candidate regions in 26 of them. The first step of our pipeline was the application of PS, which enabled us to genetically diagnose 20 index patients with novel and reported mutations in known IRD genes. This high diagnostic yield is consistent with previous work [16,49] and reinforces the high efficiency of population-based panels for this group of disorders [2]. Remarkably, the application of this first method has been crucial for the screening of families susceptible to being subjected to large-scale studies, searching genetic causes in regions not previously evaluated. 

The second step of our pipeline consisted of the use of CES to 11 cases genetically undiagnosed by PS. In four of the families, this strategy led to the detection of mutations in IRD genes not included (*FAM161A*, *MFRP* and *RP1L1*) or with uncovered regions (ORF15 of *RPGR*) in the custom panel. Detected variants in *MFRP* and *RP1L1* allowed us not only to clarify the clinical diagnosis of Families 17 and 40, but to establish new genotype–phenotype correlations by associating the posterior microphthalmos, pigmentary retinopathy syndrome with foveoschisis without optic nerve drusen [50] and fundus flavimaculatus with *MFRP* and *RP1L1* mutations, respectively. Besides, the use of a “primary” BED file containing all the regions to be studied and not only those theoretically covered by probes (“captured” BED), and the utilization of the Illumina’s high-throughput sequencing platform HiSeq3000, allowed the detection of a mutation in the ORF15 of *RPGR* (c.2655_2656del; p.(Glu886Glyfs*192)) in Family 36. This repetitive region was largely uncovered by PS and was not included in the “captured” BED file of any of the used methods since surrounding probes could not be designed. Therefore, the detected mutation could not be identified previously by PS and indeed would have been also missed during CES-data processing, as it would be considered off-target, if capture-dependent files had been used. Altogether, analysis of IRD genes from CES data has allowed the update of our personalized panel as well as the selection of appropriate cases for the study of non-IRD associated genes.

Given the large heterogeneity of rare diseases and the extensive number of functions of related genes, it is not surprising that variants in a specific gene may be associated with different human diseases [51,52]. In this regard, for unsolved cases, the analysis of CES data was extended to all genes included in the design with the aim of identifying new candidate genes. This approach resulted in the identification of a potential candidate gene (*WDFY3*) in Family 38. Functionally, WDFY3 is associated with autophagy, an essential process for retinal cells to prevent the accumulation of phototransduction effector proteins, which is described to cause retinal degeneration [53,54], and a decrease of oxidative stress in retinal epithelial cells [55]. In addition, animal models of mutations in *WDFY3* have been reported in the literature and in public databases, showing a severe eye-related phenotype in Drosophila [48]. These results, along with the expression of this gene in human retina and familial segregation suggest the involvement of *WDFY3* in the autosomal recessive IRD of this family. However, additional studies would be needed to definitely confirm the role of this gene in the etiopathogenesis of retinal disorders.

Finally, WES was carried out in five CES-negative cases. Together with STR linkage, this strategy allowed the correlation of a X-linked region with the disease in one of the families (Family 35). This region was observed in homozygous state in the affected individual with Klinefelter syndrome and in hemizygosity in the XY affected male. Therefore, a partial loss-of-function mutation [56,57,58] contained within this region could explain the intrafamilial clinical variability through an offset mechanism based on the double but insufficient gene dose in the XXY individual [59,60]. In this regard, a comprehensive analysis of the linked region was conducted and the only coding variant meeting the applied lax criteria was found in the *CITED1* gene (c.182C>T, p.(Ala61Val)). CITED1 acts as a negative regulator of *MITF* expression, which plays an essential role in the development of retinal pigmented epithelium cells [61] and is a regulator of two IRD-related genes [47,62,63,64]. However, the pathogenicity of the identified *CITED1* variant has been questioned, since it was detected in two individuals of a healthy control database (GnomAD) in hemizygous state and it is not considered as a deleterious variant for most in silico predictions tools. Of note, an oligogenic inheritance would also be consistent considering the two *PDE6B* variants (c.1345C>T, p.(Gln449*) and c.1593A>T, p(=)) also detected in both affected patients. In this light, the combination of *CITED1* and *PDE6B* mutations could lead to the development of retinal disorders, explaining both the absence of disease-phenotype in hemizygous controls and the intrafamilial variability. Even though modifier alleles and genes contributing to digenic retinal dystrophies have been previously described [65,66], an experimental demonstration would be necessary to confirm this epistatic hypothesis. 

Altogether, the comparative study of the three NGS-approaches carried out has provided us with valuable data to establish a cost-efficient diagnostic algorithm for IRD cases. In this regard, sequencing of a population-specific gene panel has been consolidated as the first approach for the genetic testing [2], showing a higher reliability, affordability, agility and flexibility than the others despite its limitations for the discovery of novel candidate genes. Of note, an updated version of the panel based on the results obtained and the current literature would potentially increase its diagnostic capacity while maintaining its cost-efficiency by continuing to be population-specific. In addition, according to our results, WES is the most helpful tool to address unsolved cases, one of the main current challenges in the diagnostic routine. This advantage of WES over CES is mainly because CES does not reach the diagnostic yield and discoverability of WES but has similar limitations regarding variant prioritization, data management and cost efforts. Therefore, this study does not support the need for an intermediate step between PS and WES. However, the use of commercial CES or exome-based panel (also called exome slices) [67] could be fitting as the only solution for genetic diagnosis centers covering multiple hereditary conditions, not aimed at discovery and for which cost is not a limiting factor. In fact, the results derived from this study may be transferred to the clinical setting by the development of a personalized local clinical exome. Importantly, since WES is still insufficient to decipher the genetic cause of some unsolved cases, understudied mutations in IRD or novel genes (e.g., deep-intronic, large rearrangements, etc.) cannot be neglected. Therefore, in agreement with published data [66,68], we hypothesize that whole genome sequencing could be the most appropriate approach following PS when its limitations in terms of processing, storage and costs are overcome. Furthermore, in populations in which deep-intronic mutations have a high contribution in hereditary conditions, a whole-gene PS approach could replace exonic PS as the first step of the algorithm. As part of the proposed workflow, it is also important to note the relevance of carrying out a completely hypothesis-free strategy, either with respect to the inheritance mode, clinical diagnosis or probes design, to maximize the diagnostic rate. However, the fact that not all cases meet the minimum pedigree and quality criteria to undergo far-reaching strategies cannot be ignored. Indeed, in this study, the complete workflow could not be applied in a total of 12 unsolved families, in which intermediate approaches such as larger panels, together with an effort to collect DNA samples from additional family members, should be carried out.

In summary, this work has allowed the development of an optimized algorithm for the diagnosis of IRD patients, defining the most optimal steps and the order in which they should be executed. Thus, this pipeline consists of PS as first diagnostic tool followed by WES in unsolved patients and a tertiary analysis (annotation, prioritization and interpretation of variants) independent of clinical and technical assumptions. Incorporating this workflow into clinical genetics practice would enhance the accuracy, feasibility and efficiency of the genetic diagnosis, allowing to expand the spectrum of these conditions. Finally, this study has proposed new candidate regions responsible for IRD, whose confirmation will help to increase knowledge about the genetic basis behind such heterogeneous diseases.

## 4. Materials and Methods

### 4.1. Patients Recruitment and Clinical Assessment

All patients included in this study were referred to the Genetics Department from different Ophthalmic Departments of Andalusian hospitals. In total, 42 families were studied in this survey (Figure 1), including 66 IRD patients and 113 healthy family members, making a total of 179 individuals. Forty-six out of 66 affected patients were sequenced following NGS methods. All affected individuals were clinically diagnosed based on the results obtained through ophthalmological evaluation, which was performed as described elsewhere [16] (Table S1). Interestingly, intrafamilial phenotypic variability was observed in one of the families (Family 35), in which both affected brothers manifested variations with respect to disease progression, being the milder affected individual also diagnosed of Klinefelter syndrome.

Genomic DNA was isolated after peripheral blood extraction using standard procedures. DNA integrity was assessed before performing each of the NGS methods using spectrophotometric and fluorometric dsDNA quantification and 1% agarose gel electrophoresis. All protocols regarding patients DNA utilization and human tissue use for immunohistochemical studies were conducted in accordance with the tenets of the Declaration of Helsinki and they were approved by the Institutional Review Boards of the University Hospital Virgen del Rocío, Seville, Spain (Project identification codes: 0974-N-15, 24th November 2015, 0141-N-19, 19th March 2019 and 1967-N-19, 24th November 2020). Written informed consent was obtained from all participants or their legal guardians prior to the study.

### 4.2. Targeted Panel Sequencing

As a first strategy, an IRD-gene panel was applied to achieve the genetic diagnosis of affected individuals and to select unsolved cases as candidates for undergoing larger-scale techniques (Figure 1). Thus, index patients belonging to 42 undiagnosed families were sequenced using a previously designed custom local panel [49] targeting commonly IRD-mutated regions in Spanish population (Table S4). Library preparation was conducted using the double capture protocol “NimbleGen SeqCap EZ Library Double Capture (version 4.2, Roche, NimbleGen, Madison, WI, USA)” and sequenced in the Illumina’s MiSeq instrument (Illumina, San Diego, CA, USA) using a v2 reagent kit (300 cycles).

### 4.3. Clinical Exome Sequencing

The selection of patients to be subjected to CES was based on three main criteria: (i) the lack of genetic diagnosis by PS; (ii) the availability of DNA from at least three relatives; and (iii) the quality of DNA samples. Thus, eleven index patients were selected (Figure 1) and captured using the SureSelect^XT^ Focused Exome design (Agilent Technologies, Santa Clara, CA, USA), consisting of exonic regions of ~4800 genes (12 Mb) that have been associated with diseases in databases as HGMD, OMIM and ClinVar. Libraries were prepared following the “SureSelect^XT^ Target Enrichment System for Illumina Paired-End Multiplexed Sequencing Library” protocol (Version B4, Agilent Technologies, Santa Clara, CA, USA). Sequencing was performed in a Hiseq3000 instrument (Illumina, San Diego, CA, USA) using a HiSeq 3000/4000 SBS Kit (300 cycles) and a HiSeq 3000/4000 PE Cluster Kit.

### 4.4. Whole-Exome Sequencing

WES was conducted in those cases that remained unsolved after CES and meeting the minimum standard quality requirements for the DNA samples from all participants. To maximize the available genomic information and facilitate the subsequent prioritization of variants, an additional affected individual, apart from the index patient, was selected when possible. Therefore, five patients (Figure 1) were picked and processed using the Roche NimbleGen SeqCap EZ MedExome Target Enrichment Kit (47 Mb) (Roche, NimbleGen, Madison, WI, USA). DNA library was performed according to “NimbleGen SeqCap EZ Library SR version 5.1” protocol. Sequencing was carried out on a NextSeq 500 instrument (Illumina, San Diego, CA, USA) with a NextSeq High-output v2 reagent kit (300 cycles).

### 4.5. Bioinformatics Analysis

Sequence data analysis was performed applying our validated pipeline [16] including open access tools and software (Table S5 [9,69,70,71,72,73,74,75,76,77,78,79,80,81,82,83,84,85,86,87,88,89,90,91,92,93]), with some modifications. Mapped reads were intersected with the primary bed file, which contained the genomic coordinates of all target regions of the design regardless of the existence of capture probes (Figure S1), using the BEDtools package (version 2.17.0). Variant calling and filtering of variants were carried out using GATK (version 3.3.0), filtering out those with low coverage (<20×) and discarding strand bias (FS > 60.0). wANNOVAR was used for SNVs and indels annotation.

The analysis of Copy-Number Variations (CNV) was conducted as previously described [16]. Cut-off points for deletions and insertions/duplications were <0.6 and >1.40, respectively. Variants were checked using DGV.

### 4.6. Prioritization of Identified Variants

**Figure 5:**
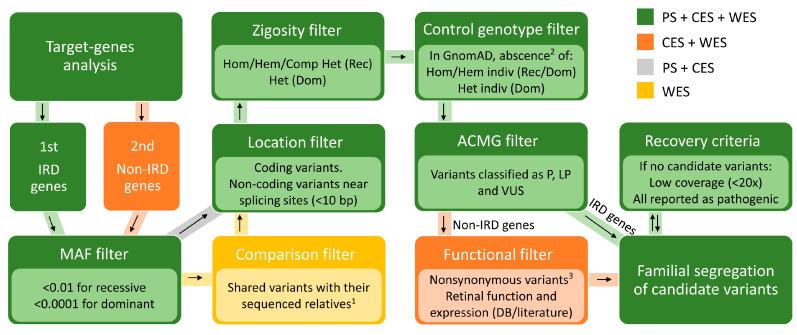
Overview of the prioritization steps performed in each strategy. Analysis of variants from panel sequencing (PS), clinical exome sequencing (CES) and whole exome sequencing (WES) using both recessive (Rec) and dominant (Dom) filters for autosomal and X-linked genes. Resulting candidate variants were subjected to familial segregation when possible. If no candidate or segregating variants were found, recovery criteria were applied to rescue pathogenic mutations that could have been filtered out. ^1^ Unshared variants were also considered to study genetic modifying factors in cases with intrafamilial phenotypic variability. ^2^ This step was only employed for variants non-previously reported as pathogenic or likely pathogenic. ^3^ Synonymous variants were not considered unless they were located in the last or first two bases of an exon. Comp het, Compound heterozygous; DB, Databases; Hem, Hemizygous; Het, Heterozygous; Hom, Homozygous; Indiv, Individuals; IRD, Inherited retinal dystrophies; LP, Likely pathogenic; P, Pathogenic; VUS, Variant of unknown significance.

Due to the huge genetic heterogeneity of IRD, detected variants were prioritized based on multiple criteria (Figure 5) and regardless of the mode of inheritance assumed for each pedigree. Firstly, according to population frequency databases (GnomAD, EVS, 1000GP and CSVS) (Table S5), variants with MAF < 0.01 for recessive approaches and MAF < 0.0001 in dominant strategies were prioritized. Additionally, non-coding variants located far away from canonical splicing sites (>10 bp) were not considered. Regarding zygosity, homozygous variants were discarded for the dominant pipeline whereas those heterozygous that are not compatible with compound heterozygosity were not considered for recessive steps. These low-frequency exonic/splicing variants previously reported as pathogenic or absent in homozygous and hemizygous state in GnomAD individuals were prioritized. Moreover, for dominant analysis, variants not present in heterozygous trait in control individuals were also highlighted. Moreover, ACMG criteria [94] were used to classify variants according to their inferred pathogenicity, discarding those with a benign or likely benign verdict. Furthermore, artifacts and *cis*-acting variants were verified, when possible, using the Integrative Genomics Viewer (IGV) (Table S5). When no causal variants were found, mutations with low coverage (<20×) and reported pathogenic variants with relatively high frequency (MAF > 0.01) were recovered.

These prioritization criteria were common for all the NGS-methods carried out, but in CES and WES additional steps were conducted (Figure 5). On the one hand, variants located in known IRD-associated genes according to RetNet (RetNet, https://sph.uth.edu/retnet, last accessed 5 May 2020) were first studied, followed by all remaining to identify variants in novel genes. Synonymous variants in genes not associated with IRD were excluded, except for those located in the last or first two bases of an exon due to their potential effect on splicing. Variants in novel candidate genes were prioritized based on gene function and expression in the literature and databases such as OMIM, Genecards, Uniprot, The Human Protein Atlas, Expression Atlas, MGI and IMPC (Table S5). Finally, WES data were compared with CES results when different affected individuals from the same family were sequenced in each of these strategies, ruling out all unshared variants located in common regions of both designs. When intrafamilial phenotypic variability was observed (Family 35), those variants not shared between patients were also highlighted in order to prevent the loss of second-site modifiers.

### 4.7. Pathogenicity Evaluation of Variants and Family Segregation Studies

Mutations that might affect the splicing process were studied by NNSPLICE, HSF and MaxEnt. Pathogenicity and conservation of *novel* nonsynonymous exonic mutations was assessed using the in silico prediction tools available in Varsome and ClustalO. Clinical significance of known variants was evaluated using ClinVar, OMIM, HGMD and LOVD. Varsome was also utilized as a support for the classification of variants based on ACMG criteria. Mutalyzer website was used to check the nomenclature of variants according to the Human Genome Variation Society (HGVS). Further details about these online resources are listed in Table S5.

Segregation analyses of candidate variants were carried out by Sanger sequencing in the case of SNVs and indels as previously described [25] and, in the case of CNVs, by qPCR using the RT^2^ SYBR Green ROX qPCR MasterMix (Qiagen, Hilden, Germany) and the 7500 Real-Time PCR system (Applied Biosystems, CA, USA). For the segregation study of the X chromosome in Family 35, 26 microsatellite markers (short tandem repeats, STR) located along the entire chromosome (Table S2) were used as reported elsewhere [95].

### 4.8. Expression and Localization Studies in Human Retina

To evaluate the expression of candidate genes, we performed qPCR using RT^2^ SYBR Green ROX qPCR MasterMix (Qiagen, Hilden, Germany) and commercial Human Retina QUICK-Clone™ cDNA (Clontech Laboratories, Inc., Mountain View, CA, USA) in an Applied Biosystems 7500HT instrument (LifeTechnologies, Carlsbad, CA, USA) according to manufacturers’ protocols. The human housekeeping gene *GAPDH* was used as loading control for the quantification of relative expression using the comparative Ct (2^-ΔΔCt^) method [96]. Brain, kidney, lung, placenta and skeletal muscle tissue cDNA (Zyagen, San Diego, CA, USA) were used for relative quantification.

Candidate genes were also studied by immunohistochemistry. For this purpose, four-micrometer-thick sections from paraffin blocks were baked for 20 min at 65 °C. Antigen retrieval was performed with a PT Link instrument (Agilent Technologies, Santa Clara, CA, USA), using EDTA buffer (97 °C, 20 min). Sections were immersed in H_2_O_2_ solution for 10 min to inactivate endogenous peroxidase activity and then covered with 1% blocking reagent (Roche, Mannheim, Germany) in PBS, to block nonspecific binding sites. Slides were incubated with primary antibodies (Abcam, ab87978 and ab84888) overnight at 4 °C in a humid chamber. Immunoreactivity was performed using horseradish peroxidase polymer conjugated secondary antibodies (Visualization reagent, Agilent Technologies, Santa Clara, CA, USA) for 1 h at R/T in a humid chamber and 3,3′-diaminobenzidine for 5 min. Slides were counterstained with hematoxylin and mounted in DPX (BDH Laboratories, Poole, UK). Sections treated with the same staining protocol omitting the primary antibody were used as negative controls. Images of the stained sections were obtained with an Olympus BX61 microscope and the cellSens Dimension software (Olympus, Center Valley, PA, USA).

### 4.9. Comparative Study of the Sequencing Strategies

To define the most cost-efficient algorithm for diagnosing IRD patients, a comparison of the different approaches was carried out, considering quality of the obtained data, amount of information generated, processing difficulty, diagnostic rate and sequencing costs. The number of variants obtained in various stages of the tertiary analysis was recorded, which also gathered information on processing difficulty. Regarding the diagnostic yield, we performed an estimation of the diagnostic rate if each approach had been applied in every index patient included in this study as a first diagnostic strategy. Besides, costs per sample were calculated for a mean coverage of 200× in PS and 100× in CES and WES and considering the maximum number of samples that can be sequenced per run according to the Sequencing Coverage Calculator (Illumina, San Diego, CA, USA) using as input parameters a 10% of duplicates and the percentage of reads mapped on target achieved in this study (96.6%, 75.3% and 74% for PS, CES and WES, respectively). In this regard, solely expenses relative to library preparation and sequencing were considered, ignoring common costs among strategies such as DNA extraction or library quality controls.

## Figures and Tables

**Table 1:** Likely pathogenic mutations found in IRD-associated genes by panel sequencing.

ID	Gene	Variants	Status	ACMG	Segr. (A/H)	Solved	Ref.
2	*ABCA4*	c.3056C>T; p.(Thr1019Met)	Het	5	Yes (1/2)	Yes	[21]
		c.3364G>A; p.(Glu1122Lys)	Het	5			[22]
3	*CHM*	c.1797C>G; p.(Cys599Trp)	Hem	3	No	Yes	This study
4	*ABCA4*	c.3287C>T; p.(Ser1096Leu)	Het	4	No	Yes	[23]
		c.466A>G; p.(Ile156Val)	Het	3			[24]
6	*EYS*	c.4451G>A; p.(Trp1484*)	Het	5	No	Yes	[25]
		c.5928-2A>G; r.spl	Het	5			[26]
10	*USH2A*	c.2299delG; p.(Glu767Serfs*21)	Het	5	Yes (2/5)	Yes	[27]
		c.13374delA; p.(Glu4458Aspfs*3)	Het	5			[28]
12	*PDE6B*	c.2193+1G>A; r.spl	Het	5	Yes (2/2)	Yes	[29]
		c.1572delC; p.(Tyr525Thrfs*50)	Het	5			This study
13	*USH2A*	c.2276G>T; p.(Cys759Phe)	Het	5	No	Yes	[30]
		c.13531G>A; p.(Ala4511Thr)	Het	3			[31]
14	*WHRN*	c.1417-8G>A; r.(spl?)	Hom	3	Yes (2/4)	No	ClinVar (45653 ^†^)
15	*USH2A*	c.12546T>G; p.(Asn4182Lys)	Het	3	No	Yes	This study
		c.13979C>G; p.(Pro4660Arg)	Het	3			GnomAD
19	*ABCA4*	c.5882G>A; p.(Gly1961Glu)	Het	4	Yes (3/3)	Yes	[32]
		c.700C>T; p.(Gln234*)	Het	5			[33]
21	*USH2A*	c.920_923dup; p.(His308Glnfs*16)	Het	5	Yes (2/6)	Yes	[34]
		c.2276G>T; p.(Cys759Phe)	Het	5			[30]
23	*ABCA4*	c.3386G>T; p.(Arg1129Leu)	Het	5	Yes (1/4)	Yes	[35]
		c.700C>T; p.(Gln234*)	Het	5			[33]
24	*ABCA4*	c.3386G>T; p.(Arg1129Leu)	Het	5	No	Yes	[35]
		c.(2382+1_2383-1)_(2587+1_2588-1)del; p.? (Deletion of exon 16)	Het	4			This study
25	*BBS10*	c.273C>G; p.(Cys91Trp)	Hom	5	Yes (2/2)	Yes	[36]
26	*CHM*	c.83C>G; p.(Ser28*)	Hem	5	Yes (1/5)	Yes	[37]
28	*PDE6B*	c.1107+3A>G; r.(spl?)	Het	4	No	Yes	[38]
		c.1969A>G; p.(Ile657Val)	Het	3			This study
29	*PROM1*	c.(1002+1_1003-1)_(1454+1_1455-1)del; p.? (Deletion of exons 9-12)	Hom	4	Yes (3/1)	Yes	This study
30	*USH2A*	c.2276G>T; p.(Cys759Phe)	Het	5	Yes (1/1)	Yes	[30]
		c.6967C>T; p.(Arg2323*)	Het	5			[39]
31	*PDE6B*	c.1923_1969delinsTCTGGG; p.(Asn643Glyfs*29)	Hom	5	Yes (1/5)	Yes	[38]
33	*USH2A*	c.2276G>T; p.(Cys759Phe)	Het	5	Yes (2/2)	Yes	[30]
		c.14011G>T; p.(Glu4671*)	Het	5			[16]
34	*ABCA4*	c.5714+5G>A; r.(spl?)	Het	5	Yes (2/3)	Yes	[40]
		c.223T>G; p.(Cys75Gly)	Het	4			[22]
35	*PDE6B*	c.1345C>T, p.(Gln449*)	Het	5	Yes (2/3)	No	dbSNP ^‡^
		c.1593A>T, p.(=)	Het	4			This study

Hem, hemizygosity; Het, heterozygosity; Hom, homozygosity; ID, family identifier; Ref., References; Segr., familial segregation studies (A/H, affected individuals/healthy individuals). ^†^ This ClinVar entry classifies the variant as conflicting interpretation of pathogenicity since both a likely benign (dated 2011) and unknown significance (dated 2017) interpretations have been submitted. ^‡^ dbSNP identifier: rs772166846.

**Table 2:** Likely pathogenic mutations detected in IRD genes by clinical exome sequencing.

ID	Gen	Variants	Status	ACMG	Segr. (A/H)	Solved	Ref.
17	*MFRP*	c.498delC; p.(Asn167Thrfs*25)	Hom	5	Yes (3/2)	Yes	[41]
36	*RPGR*	c.2655_2656del; p.(Glu886Glyfs*192)	Hem	5	Yes (2/5)	Yes	[42]
37	*FAM161A*	c.1309A>T; p.(Arg437*)	Hom	5	Yes (1/4)	Yes	[43]
40	*RP1L1*	c.5821C>T; p.(Gln1941*)	Het	5	Yes (1/6)	Yes	ClinVar (361237)
		c.3412A>G; p.(Lys1138Glu)	Het	3			This study

Hem, hemizygosity; Het, heterozygosity; Hom, homozygosity; Ref., references; Segr., familial segregation studies (A/H, affected individuals/healthy individuals).

**Table 3:** Summary of data related to candidate genes and identified variants.

**Family**		38		35
**Applied strategy**		CES		WES
**Sequenced individual**		II:1		II:2
**Gene**		*WDFY3*		*CITED1*
**Variant**		M1	M2	M3
**Status**		Het	Het	Hem
**Segr. (A/H)**		Yes (1/3)		Yes (2/3)
**Reference**		Novel	Novel	Novel
**GnomAD GC (Het/Hem/Hom)**		NA	2/0/0	9/2/0
**ACMG**		VUS	VUS	VUS
**In silico prediction**		CIP	PP	PB
**Conserv.**	**ClustalO**	V	V	P
	**PhyloP**	V (7.49)	V (4.22)	NC
	**PhastCons**	P (0.998)	P (0.995)	P (0.666)
	**GERP**	C (5.5)	C (5.8)	NC (1.3)
**Retinal expression**		Yes		Yes
**Reported function**		Scaffolding protein in autophagy [44]		Transcriptional co-regulator [45] of *MITF* [46], associated with the pigmentation process and regulator of *RLBP1* and *RDH5* IRD- genes [47]
**Previously associated with IRD**		No		No
**Animal models**	**DB**	Perinatal lethality in mouse.		Placental disorders and neonatal lethality.
	**Ref.**	Eye-related phenotype in Drosophila [48]		NA

C, conserved; CES, clinical exome sequencing; CIP, conflicting interpretation of pathogenicity; Conserv., conservation; DB, databases; GC, genotype count; Hem, hemizygous; Het, heterozygous; IRD, inherited retinal dystrophies; M1, *WDFY3* c.2891G>A, p.(Arg964Lys); M2, *WDFY3* c.10465C>T, p.(Arg3489Cys); M3, *CITED1* c.182C>T, p.(Ala61Val); NA, not available; NC, not conserved; P, primates; PB, possibly benign; PP, possibly pathogenic; Ref., references; Segr., familial segregation studies (A/H, affected individuals/healthy individuals); V, vertebrates; VUS, variant of unknown significance; WES, whole exome sequencing.

## Supplementary Materials

The following are available online at https://www.mdpi.com/1422-0067/21/24/9355/s1, Table S1: Clinical findings in the 46 sequenced patients. “Typical of RP” refers to pigmentary deposits, optic disc pallor and attenuation of retinal vessels. When more than one affected individual was sequenced per family (Families 22, 35, 41 and 42), only clinical manifestations of the index patient are shown, except when intrafamilial phenotypic variability was observed. * diagnosed cases. AD, autosomal dominant inheritance; AR, autosomal recessive inheritance; BBS, Bardet–Biedl syndrome; BCVA, best corrected visual acuity; CES, clinical exome sequencing; CHM, choroideremia; CORD, cone–rod dystrophy; ERG, electroretinography; F, female; FF, fundus flavimaculatus; M, male; MFT, microphthalmia; NR, not recordable; PS, panel sequencing; RP, retinitis pigmentosa; sRP, simplex retinitis pigmentosa; STGD, Stargardt disease; USH, Usher syndrome; WES, whole exome sequencing; XL, X-linked inheritance. Table S2: STR markers used in the linkage analysis of the entire X-chromosome in Family 35. All used STR markers, along with their X-chromosome coordinates and their allele length for each individual of the family, are shown. Sex chromosomes are also indicated for clarity reasons. Table S3: Comparison of sequencing and analysis parameters among the different applied strategies. The estimated diagnostic rate for each of the approaches used was calculated regardless enrichment and sequencing limitations and considering a total of 24 (omitting cases with candidate genes) or 26 (including cases with candidate genes) diagnosed cases, as appropriate. Rare variants refer to those with a MAF < 0.01. * Cost calculated using a MiSeq instrument with a v2 MiSeq Reagent Kit. † Cost calculated using a NextSeq 500 instrument with a v2 NextSeq 500/550 High Output Reagent Kit. Table S4: List of genes included in the capture IRD panel and relevant phenotypes for this study. This panel includes 66 known retinal genes and 2 former candidate loci (unpublished data). Non-coding positions of *CEP290*, *OFD1* and *USH2A* have been included in this study for the screening of reported intronic pathogenic mutations: * 1 = c.2991+1655A>G; * 2 = c.935+706A>G; * 3 = c.7595-2144 A>G. ACHM, Achromatopsia; AD, Autosomal dominant; AR, Autosomal recessive; BBS, Bardet–Biedl syndrome; CD, Cone dystrophy; CRD, Cone–rod dystrophy; FEVR, Familial exudative vitreoretinopathy; LCA, Leber congenital amaurosis; RP, Retinitis pigmentosa; STGD, Stargardt disease; USH, Usher syndrome; XL, X-linked. Table S5: Bioinformatics resources used in this study. Full name of each tool, as well as its acronym, web address (URL) and reference (Ref.), is listed when possible. Figure S1: Visualization of the reads sequenced by PS and CES in the ORF15 of RPGR and differences between BED files in this repetitive region. IGV snapshot showing the ORF15-mapped reads from clinical exome sequencing (CES) and gene panel sequencing (PS) in the index patient of Family 36. This region was largely uncovered by PS as no reads were observed. However, CES allowed the identification of a small deletion (c.2655_2656del; p.(Glu886Glyfs*192)) in this region, whose location is shown zoomed in. Genomic positions included in the capture and primary BED files are indicated by thick blue lines. According to RefSeq data, primary BED file fully contains the ORF15, whereas the capture BED file presents a gap of around 1 kb. ChrX, Chromosome X.

## Abbreviations

CES	Clinical Exome Sequencing
CNV	Copy-Number Variations
GnomAD	Genome Aggregation Database
IRD	Inherited Retinal Dystrophies
NGS	Next Generation Sequencing
RP	Retinitis Pigmentosa
STR	Short Tandem Repeats
VUS	Variants of Unknown Significance
WES	Whole Exome Sequencing
WGS	Whole Genome Sequencing
