# Supplementary material for: A Multi-Strategy Sequencing Workflow in Inherited Retinal Dystrophies: Routine Diagnosis, Addressing Unsolved Cases and Candidate Genes Identification
Source: Int J Mol Sci. 2020 Dec 8;21(24):9355. doi: 10.3390/ijms21249355 (PMC7763277; doi:10.3390/ijms21249355)

SUPPLEMENTARY INFORMATION

A multi-strategy sequencing workflow in inherited retinal dystrophies: routine diagnosis, addressing unsolved cases and candidate genes identification.

Marta Martín-Sánchez[†], Nereida Bravo-Gil[†], María González-del Pozo, Cristina Méndez-Vidal, Elena Fernández-Suárez, Enrique Rodríguez-de la Rúa, Salud Borrego and Guillermo Antiñolo[*]

[†] Equally contributing authors

* Corresponding author

Figure S1. Visualization of the reads sequenced by PS and CES in the ORF15 of *RPGR* and differences between BED files in this repetitive region. IGV snapshot showing the ORF15-mapped reads from clinical exome sequencing (CES) and gene panel sequencing (PS) in the index patient of family 36. This region was largely uncovered by PS as no reads were observed. However, CES allowed the identification of a small deletion (c.2655_2656del; p.(Glu886Glyfs*192)) in this region, whose location is shown zoomed in. Genomic positions included in the capture and primary BED files are indicated by thick blue lines. According to RefSeq data, primary BED file fully contains the ORF15 whereas the capture BED file presents a gap of around 1 kb. ChrX: Chromosome X.

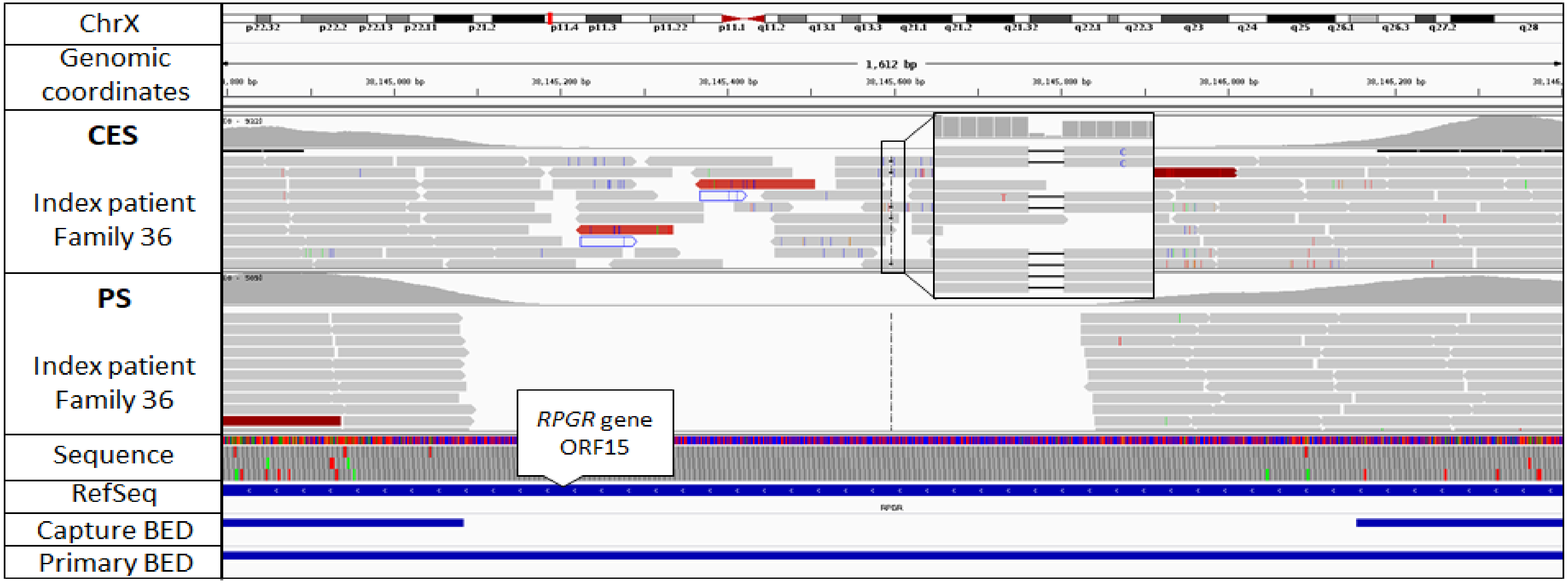

Supplement: Supplementary file 1 [file ijms-21-09355-s001.zip › ijms-1024951-suppl/Figure S1.pdf]
