# Supplementary material for: A Multi-Strategy Sequencing Workflow in Inherited Retinal Dystrophies: Routine Diagnosis, Addressing Unsolved Cases and Candidate Genes Identification
Source: Int J Mol Sci. 2020 Dec 8;21(24):9355. doi: 10.3390/ijms21249355 (PMC7763277; doi:10.3390/ijms21249355)
Supplement: Supplementary file 1 [file ijms-21-09355-s001.zip › ijms-1024951-suppl/Table S5.docx]

**SUPPLEMENTARY INFORMATION**

**A multi-strategy sequencing workflow in inherited retinal dystrophies: routine diagnosis, addressing unsolved cases and candidate genes identification.**

Marta Martín-Sánchez^†^, Nereida Bravo-Gil^†^, María González-del Pozo, Cristina Méndez-Vidal, Elena Fernández-Suárez, Enrique Rodríguez-de la Rúa, Salud Borrego and Guillermo Antiñolo^*^

^†^ Equally contributing authors

* Corresponding author

**Supplementary Table 5. Bioinformatics resources used in this study.** Full name of each tool, as well as its acronym, web address (URL) and reference (Ref.), is listed when possible.

| **Tool name** | **URL** | **Ref.** |
| --- | --- | --- |
| 1000 Genome Project (1000GP) | https://www.ncbi.nlm.nih.gov/variation/tools/  1000genomes/ | [69] |
| BEDtools | - | [70] |
| Burrows-Wheeler Aligner (BWA) | - | [71] |
| ClinVar | https://www.ncbi.nlm.nih.gov/clinvar/ | [72] |
| ClustalO | https://www.ebi.ac.uk/Tools/msa/clustalo/ | [73] |
| Collaborative Spanish Variant Server (CSVS) | http://csvs.babelomics.org/ | [9] |
| Database of Genomic Variants (DGV) | http://dgv.tcag.ca/dgv/app/home | [74] |
| Exome Variant Server (EVS) | https://evs.gs.washington.edu/EVS/ | - |
| Expression Atlas | https://www.ebi.ac.uk/gxa/home | [75] |
| Genecards | https://www.genecards.org/ | [76] |
| Genome Aggregation Database (GnomAD) | https://gnomad.broadinstitute.org/ | [77] |
| Genome Analysis Toolkit software (GATK) | - | [78] |
| HSF | http://www.umd.be/HSF | [79] |
| Integrative Genomics Viewer (IGV) | - | [80] |
| International Mouse Phenotyping Consortium (IMPC) | https://www.mousephenotype.org/ | [81] |
| Leiden Open Variation Database (LOVD) | https://www.lovd.nl/ | [82] |
| MaxEntScan | http://www.umd.be/HSF | [83] |
| Mouse Genome Informatics (MGI) | http://www.informatics.jax.org/ | [84] |
| Mutalyzer | https://mutalyzer.nl/ | [85] |
| NNSPLICE | http://www.fruitfly.org/seq_tools/splice.html | [86] |
| Online Mendelian Inheritance in Man (OMIM) | https://www.omim.org/ | [87] |
| PICARD | https://broadinstitute.github.io/picard/ | - |
| Retinal Information Network (RetNet) | https://sph.uth.edu/retnet/home.htm | - |
| Single Nucleotide Polymorphism Database (dbSNP) | https://www.ncbi.nlm.nih.gov/snp/ | [88] |
| The Human Gene Mutation Database (HGMD) | http://www.hgmd.cf.ac.uk/ac/index.php | [89] |
| The Human Protein Atlas | https://www.proteinatlas.org/ | [90] |
| Uniprot | https://www.uniprot.org/ | [91] |
| Varsome (ACMG implementation tool) | https://varsome.com/ | [92] |
| wANNOVAR | http://wannovar.wglab.org/ | [93] |
